# Supplementary material for: A bacterial cell factory converting glucose into scyllo-inositol, a therapeutic agent for Alzheimer’s disease
Source: Commun Biol. 2020 Mar 2;3:93. doi: 10.1038/s42003-020-0814-7 (PMC7052218; doi:10.1038/s42003-020-0814-7)
Supplement: Supplementary file 1 — Supplementary Information [file 42003_2020_814_MOESM1_ESM.pdf]

***rpsO*-SD**

ggtaactggggctaaattatgat***tggagg***tgaaacagg*ATG*TCTGAACATCAATCTCTTC
CTGCTCCTGAAGCTTCTACAGAAGTTCGTGTTGCTATCGTTGGCGTTGGCAA
CTGCGCTTCTTCTCTTGTTCAAGGCGTTGAATACTACTACAACGCTGATGAT
ACATCTACAGTTCCTGGCCTTATGCATGTTCGTTTCGGCCCTTACCATGTTCG
TGATGTTAAATTCGTTGCTGCTTTCGATGTTGATGCTAAAAAAGTTGGCTTC
GATCTTTCTGATGCTATCTTCGCTTCTGAAAACAACACAATCAAAATCGCTG
ATGTTGCTCCTACAAACGTTATCGTTCAACGTGGCCCTACACTTGATGGCAT
CGGCAAATACTACGCTGATACAATCGAACTTTCTGATGCTGAACCTGTTGAT
GTTGTTCAAGCTCTTAAAGAAGCTAAAGTTGATGTTCTTGTTTCTTACCTTCC
TGTTGGCTCTGAAGAAGCTGATAAATTCTACGCTCAATGCGCTATCGATGCT
GGCGTTGCTTTCGTTAACGCTCTTCCTGTTTTCATCGCTTCTGATCCTGTTTG
GGCTAAAAAATTCACAGATGCTCGTGTTCCTATCGTTGGCGATGATATCAAA
TCTCAAGTTGGCGCTACAATCACACATCGTGTTCTTGCTAAACTTTTCGAAG
ATCGTGGCGTTCAACTTGATCGTACAATGCAACTTAACGTTGGCGGCAACA
TGGATTTCCTTAACATGCTTGAACGTGAACGTCTTGAATCTAAAAAAATCTC
TAAAACACAAGCTGTTACATCTAACCTTAAACGTGAATTCAAAACAAAAGAT
GTTCATATCGGCCCTTCTGATCATGTTGGCTGGCTTGATGATCGTAAATGGG
CTTACGTTCGTCTTGAAGGCCGTGCTTTCGGCGATGTTCCTCTTAACCTTGA
ATACAAACTTGAAGTTTGGGATTCTCCTAACTCTGCTGGCGTTATCATCGAT
GCTGTTCGTGCTGCTAAAATCGCTAAAGATCGTGGCATCGGCGGCCCTGTT
ATCCCTGCTTCTGCTTACCTTATGAAATCTCCTCCTGAACAACTTCCTGATGA
TATCGCTCGTGCTCAACTTGAAGAATTCATCATCGGC*TAATAA*gagtaatagtat
ggttttaaacgagacccctgtgggtctcgtttttttgtttgcttatctttttcttgtataata

***tufA*-terminator**

Supplementary Figure 1: Sequence of *ino1* of *M. tuberculosis* optimized for translation in *B. subtilis*. The nucleotide sequence in upper-case letters is the coding region; the translation start codon and termination codons are in italics. Those in lower-case underlined letters are the *rpsO* ribosome binding site (*rpsO*-SD) and the *tufA*-terminator attached at the head and tail, respectively.

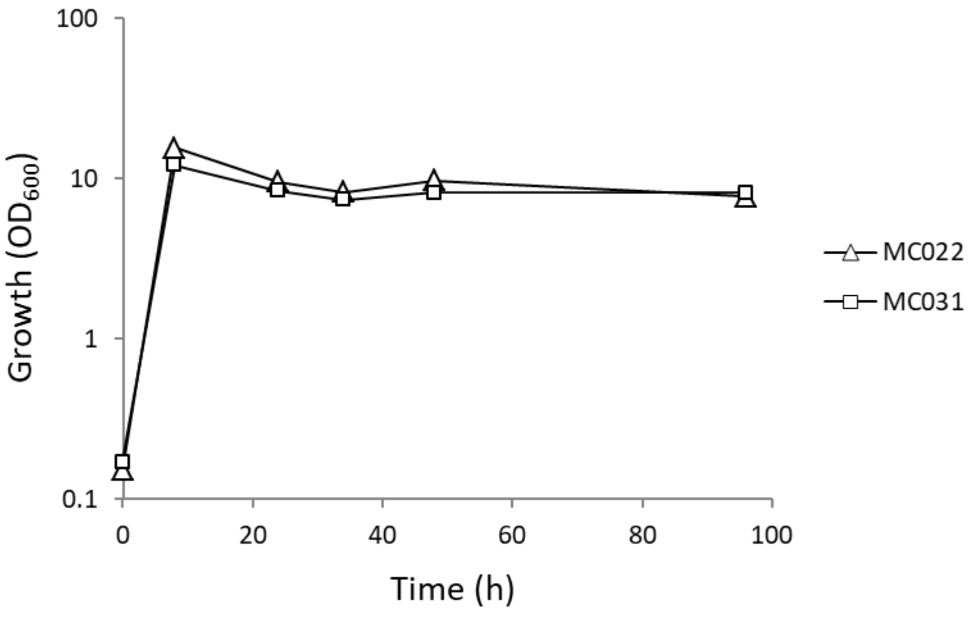

Supplementary Figure 2: Growth curves of strains MC022 [Δ*iolABCDEF* Δ*iolHIJ* Δ*iolX* Δ*iolR* *amyE*::(P*rpsO-iolG-iolW-iolT kan*) *pbuE*::(P*rpsO-ino1Mt-His*$_6$ *cat*), triangles] and MC031 [Δ*iolABCDEF* Δ*iolHIJ* Δ*iolX* Δ*iolR* *amyE*::(P*rpsO-iolG-iolW-iolT kan*) *pbuE*::(P*rpsO-ino1Mt-His*$_6$ *cat*) *epr*::(P*rpsO-glcP-glcK ble*), squares]. MC022 and MC031 were grown under the conditions for SI production as in Fig. 4. A set of representative results from three independent experiments is shown.

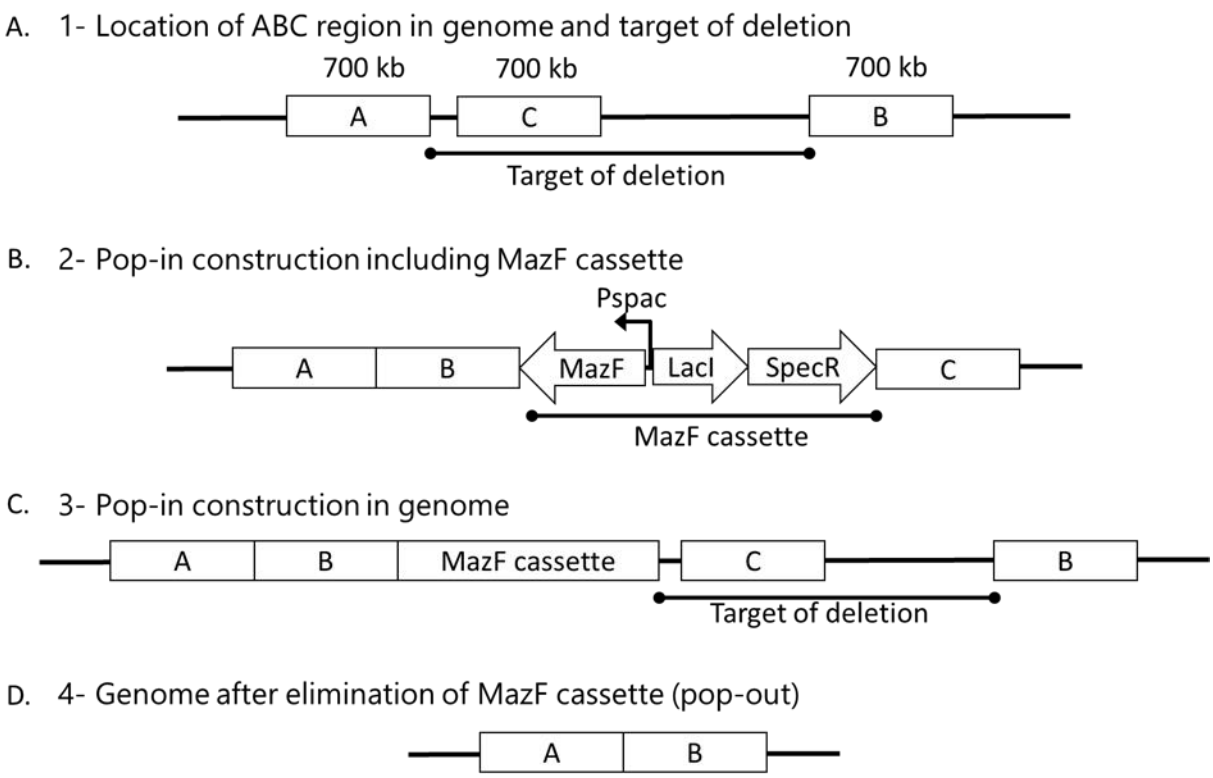

Supplementary Figure 3: A schematic representation of the marker-free deletion technique.

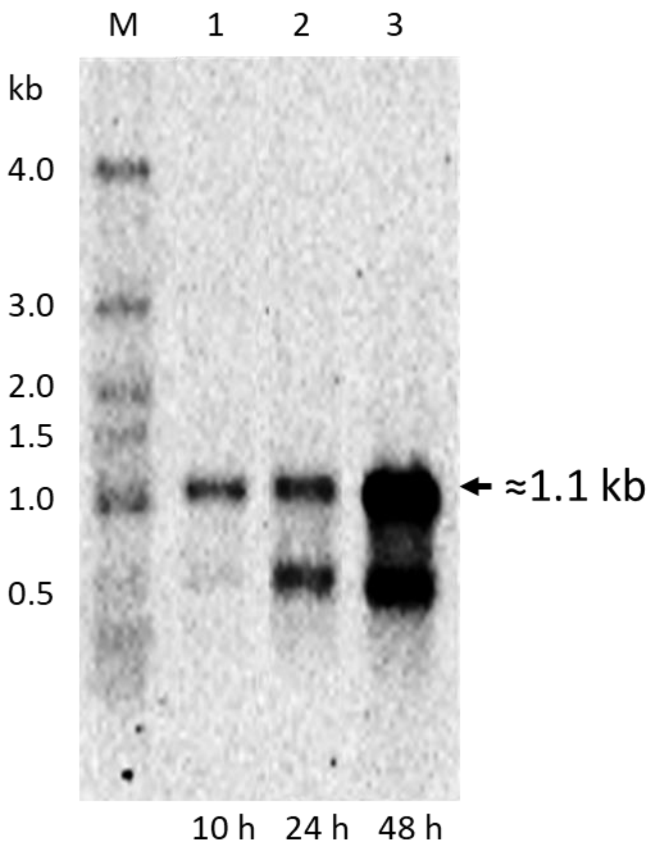

Supplementary Figure 4: The original gel image of Fig. 2a. The *ino1* transcript in *B. subtilis* TK002 was detected at the expected size (1.1 kb) by Northern blot analysis and accumulated as the cells grew after culturing for 10, 24, and 48 h (lanes 1, 2, and 3, respectively; each lane contained 30 μg of the RNA extract.). The arrowhead indicates the position of bands for the full-length transcript. The lane M contained size markers.

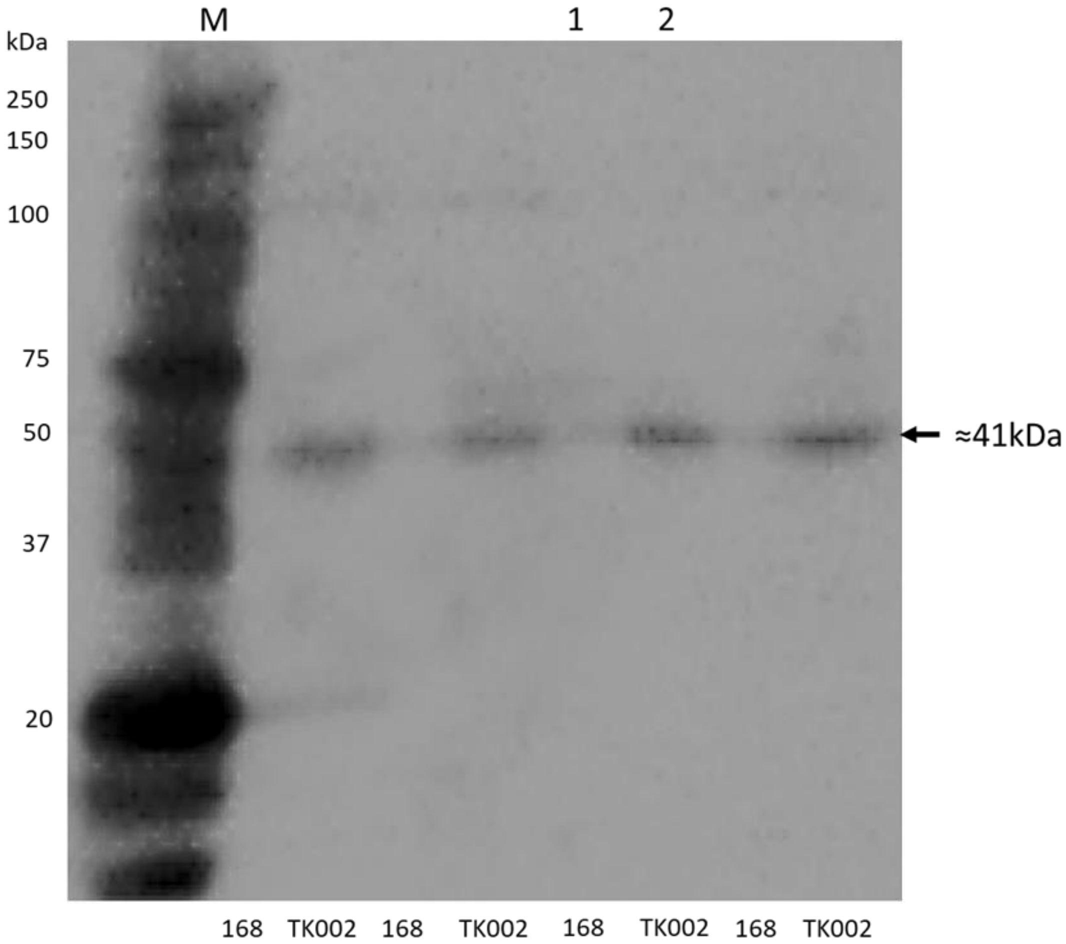

Supplementary Figure 5: The original gel image of Fig. 2b. The protein extracts of *B. subtilis* 168 and TK002 (50 μg per lane) were subjected to SDS-PAGE followed by the Western blot analysis with the anti-His-tag antibody to detect MI1PS as a C-terminal His-tag fusion. The MI1PS protein was detected at the expected size (41 kDa) in strain TK002 (lane 2) but not in strain 168 (lane 1). The arrowhead indicates the position of the band for the protein. The lane M contained size markers.

Supplementary Table 1: List of strains used in this study

Name	Relevant genotype	Source of reference
168	*trpC2*	Laboratory stock
KS001	Δ*iolABCDEFGHIJ* Δ*iolX* Δ*iolR* (168 background)	This study
KU302	*amyE*::(P*rpsO-iolG-iolW-iolT cat*) (MYI04 background)	15
MC001	*amyE*::(P*rpsO-ino1Mt-*His_6 *cat*) (KS001 background)	This study
MC010	*pbuE*::pMutin2(*erm*) (KS001 background)	This study
MC011	*pbuE*::pMutin2(*erm*) (MC001 background)	This study
MC020	*amyE*::(P*rpsO-iolG-iolW-iolT kan*) (KU302 background)	This study
MC021	*pbuE*::(P*rpsO-ino1Mt-*His_6 *cat*) (168 background)	This study
MC022	*pbuE*::(P*rpsO-ino1Mt-*His_6 *cat*) (MC020 background)	This study
MC030	*epr*::(P*rpsO-glcP-glcK ble*) (168 background)	This study
MC031	*epr*::(P*rpsO-glcP-glcK ble*) (MC022 background)	This study
MYI04	Δ*iolABCDEF* Δ*iolHIJ* Δ*iolX* Δ*iolR* (168 background)	18
TK001	*amyE::*(P*rpsO-ino1Mt cat*) (168 background)	This study
TK002	*amyE::*(P*rpsO-ino1Mt-*His_6 *cat*) (168 background)	This study
TK003	*amyE*::(P*ybfK-ino1Mt-*His_6 *cat*) (168 background)	This study
TM0310	*aprE*::(*spc lacI* P*spac-mazF*)	41
YDHLd	*pbuE*::pMutin2(*erm*) (168 background)	NBRP

Supplementary Table 2: List of primers used in this study

Primer	Sequence (5'–3')
AmyAB	ccaaatcatatttagccccagttacc
AmyAF	ccttccagggtatgtttctc
AmyBB	ttaacaaaattctccagtcttcacatcg
AmyBF	cgatcagaccagtttttaatttgtg
cmino1B	attccaaactggacacatgg
cmino1F	ttccccatcggtgatgt
DIGinoMtB	taatacgactcactatagggatatcatcgccaacgatagg
DIGinoMtF	aagttggcttcgatctttct
DiolGAF	ccggagaaacagcttcat
DiolGAR	agttcaaaactaaactaaagagaccaga
DiolGBF	tctggtctctttagtttagttttgaactttattatatatttatgttacgtaaagattc
DiolGBR	gcttgagtcaattccgctgtcgaggctgttttcttcttatgc
DiolGCF	ctgattgggtaggatccccggttgtaaaagattgatagaattccggt
DiolGCR	tatgtgtttactaatgaaggaggcaat
EprAB	gctcagttaattctttgatgccatgaaacgccttaacaaagtga
EprAF	cctttgctgaagaaaagtcg
EprBB	ggcaagctcgttaaaatcaa
EprBF	gcaatcgccctaatatatgccattgctcaaagttgtcag
GlcKB	ccgaatagcaaaaaactggcttaacctgagcacaacaaa
GlcKF	tcaaaaacacagtagaaaacgtatagctaaggtgataaaaatggacg
GlcPB	tatacgttttctactgtgtttttga
GlcPF	ggtaactggggctaaatatgatttggactttcctgctttttcatatagg
hister	catcaccaccatcatcaccatcatcac
ino1B	cacaaattaaaaactggtctgatcgtattatacaagaaaaagataagcaaacaaaaaacgagaccc
ino1F	ggtaactggggctaaatatgatttggggtaactggggctaaattatgat

inohis	ggtgatgatggtggtgatggccgatgatgaattcttcaag
MazFF	cgacagcggaattgactcaagc
MazFR	cggggatcctacccaatcag
pbuEAB	acatcaccgatggggaacattatgttcttactgccgc
pbuEAF	cattcagactcctcaacaca
pbuEBB	ctttttcccttctttataggcg
pbuEBF	ccatgtgtccagtttggaatatgttttgataaataaaaaaaaatcctgatt
PhleoB	catatattagggcgattgc
PhleoF	ccagttttttgctattcgg
PrpsOA	atggcatcaaagaattaactgagc
PrpsOB	ccaaatcatatttagccccagttacc
PybfKB	ccaaatcatatttagccccagttaccgattgatataaaggataacgcccc
PybfKF	cccactttatccaattttcgtttgcatgccattccattagtcttcaaag
