# Supplementary material for: A bacterial cell factory converting glucose into scyllo-inositol, a therapeutic agent for Alzheimer’s disease
Source: Commun Biol. 2020 Mar 2;3:93. doi: 10.1038/s42003-020-0814-7 (PMC7052218; doi:10.1038/s42003-020-0814-7)
Supplement: Supplementary file 2 — Descriptions of Additional Supplementary Files [file 42003_2020_814_MOESM2_ESM.pdf]

The raw data for graphs are supplied as additional supplementary files in Excel format as follows:

Supplementary Data 1.xlsx (for Fig. 3a)

Supplementary Data 2.xlsx (for Fig. 3b)

Supplementary Data 3.xlsx (for Fig. 4)
